# Maternal autistic traits and antenatal pain by cross-sectional analysis of the Japan Environment and Children’s Study

**DOI:** 10.1038/s41598-023-32945-2

**Published:** 2023-04-13

**Authors:** Keiko Yamada, Takashi Kimura, Meishan Cui, Eizaburo Tanaka, Yasuhiko Kubota, Satoyo Ikehara, Hiroyasu Iso

**Affiliations:** 1 Public Health, Department of Social Medicine, Osaka University Graduate School of Medicine, 2-2 Yamadaoka, Suita, Osaka 565-0871 Japan; 2 Pain Medicine, Juntendo University Graduate School of Medicine, Tokyo, Japan; 3 Department of Anesthesiology and Pain Medicine, Juntendo University Faculty of Medicine, Tokyo, Japan; 4 Department of Public Health, Hokkaido University Graduate School of Medicine, Sapporo, Hokkaido Japan; 5 Hyogo Institute for Traumatic Stress, Kobe, Hyogo Japan; 6 Osaka Center for Cancer and Cardiovascular Diseases Prevention, Osaka, Japan; 7 Department of Public Health Medicine, Faculty of Medicine, University of Tsukuba, Tsukuba, Ibaraki Japan; 8 Institute for Global Health Policy Research (iGHP), Bureau of International Health Cooperation, National Center for Global Health and Medicine, Tokyo, Japan

**Keywords:** Medical research, Risk factors, Signs and symptoms

## Abstract

The aim of cross-sectional study was to investigate whether the presence of autistic traits in pregnant women was positively associated with the prevalence and severity of antenatal pain. We analyzed 89,068 pregnant women from a Japanese national birth cohort cross-sectionally. Autistic traits were assessed using the Japanese version of the Autism-Spectrum Quotient short form (AQ-10-J). Antenatal pain was measured using the SF-8 bodily pain item (SF-8-Pain). Antenatal pain in the second to third trimester during pregnancy was categorized into three groups: without pain, mild pain, and moderate-to-severe pain. Participants were divided into eight groups by AQ-10-J score: seven consecutive scoring groups (scores 0–6), and those above the cut-off (≥ 7) for probable autistic spectrum disorders. Odds ratios (OR) for the prevalence of mild and moderate-to-severe pain were calculated for each AQ-10-J scoring group (reference: without pain group) using multinominal logistic regression analysis. Autistic traits were positively associated with mild and moderate-to-severe pain in a dose–response manner, but the association with moderate-to-severe pain was strongest. Fully-adjusted ORs (95% confidence intervals) for moderate-to-severe pain were: 1.01 (0.91–1.13) for 1 point, 1.13 (1.02–1.25) for 2 points, 1.16 (1.04–1.29) for 3 points, 1.20 (1.07–1.34) for 4 points, 1.23 (1.09–1.40) for 5 points, 1.27 (1.10–1.47) for 6 points, and 1.24 (1.05–1.46) for ≥ 7 points (AQ-10-J cut-off). We identified an association between maternal autistic traits and antenatal pain. Maternal autistic traits may need to be considered when addressing antenatal pain during healthcare for expectant mothers.

## Introduction

More than two-thirds of pregnant women experience antenatal pain, including lower back pain, pelvic pain, and headache^1,2^. The International Association for the Study of Pain stated that “pain is always personal experience that is influenced to varying degrees by biological, psychological, and social factors”^3^. Therefore, each pregnant woman may feel pain differently. Although differences in the biological mechanism between antenatal pain and persistent pain other than in the pregnancy period remain unclear, the pregnancy period is an important phase for women that must be considered in public health. Antenatal pain affects household tasks, sick leave, relationships with others, psychological health, and quality of life during pregnancy^4,5^. Antenatal pain also increases the risk for depression and maternal bonding disorder after delivery^6^. Furthermore, antenatal pain has a long-term influence on women’s health. For example, pregnancy-related lumbopelvic pain has been associated with anxiety and depressive symptoms for up to 11 years after delivery^7^. Although antenatal pain has a major impact on women’s health, it is difficult to control using pharmacological and non-pharmacological treatments^8^. A recent scoping review reported that risk factors for pregnancy-related pelvic girdle pain were young age, low educational level, overweight/obesity, no pre-pregnancy exercise, physically demanding work, history of childbirth, use of a progestin-intrauterine device, previous back trauma/disease, stress, depression, and anxiety^9^. However, there are additional risk factors for antenatal pain that remain to be explored.

Alteration of pain responses among individuals with autistic traits such as autistic spectrum disorder (ASD) have been reported, including inconsistent observations of both hyposensitivity and hypersensitivity to pain^10^. Therefore, women with autistic traits may have different experiences and expressions of antenatal pain than women without such traits. A clinical report noted that individuals with ASD had increased tolerance to pain stimulation^11^. A previous study that used quantitative sensory testing reported that individuals with ASD had higher mechanical pain and light touch detection thresholds and greater ranges of extreme scores compared with those without ASD^12^. Other research found that people with ASD had different central responses to pleasant and unpleasant stimulation than those without ASD^13^. In addition, compared with those without ASD, individuals with ASD were reported to show stronger responses to heat pain at high temperature but indifferent responses to low thermal stimulation^14^. Pain response among individuals with ASD may therefore depend on the context and type of stimulation. Moreover, a recent study that explored pain response in individuals with ASD, suggested that alteration of neural networks among individuals with ASD (detected using neuroimaging) contributed to pain coping^15^.

However, these observations were related to pain caused by mechanical stimulation (e.g., evoked pain), which differs from spontaneous pain. Antenatal pain is considered spontaneous pain rather than evoked pain. In relation to spontaneous pain, children and adolescents with ASD showed a two-fold higher prevalence of repeated or chronic physical pain^16^, and approximately 14% of children and adolescents who visited pain clinics were diagnosed with comorbid ASD^17^. However, no previous observational study has examined associations between adult autistic traits and the experience of antenatal pain.

Individuals with ASD often have comorbid anxiety and depression^18,19^. Pain experience may also be amplified by psychological stress and negative emotions, which are regulated by interoceptive pathways in the central nervous system (e.g., the anterior insular cortex and anterior cingulate cortex)^20^. A previous qualitative study reported that pregnant women with ASD tended to experience anxiety, depression, other psychiatric disorders, and sleep difficulties as well as antenatal pain because of their difficulty in social communication with medical staff^21^. Therefore, we also hypothesized that there was an interaction of comorbid psychological risk factors (e.g., anxiety disorder, depression, and schizophrenia) in the association between maternal autistic traits and antenatal pain.

Autistic traits are clinically defined in gradations, and the presence of extreme autistic traits is diagnosed as ASD. Individuals with mild autistic traits are broadly distributed across the population (i.e., broader autism phenotype)^22,23^. In addition, many ASD cases remain undiagnosed. Previous studies have reported cases of persistent pain explained by undiagnosed ASD^24,25^. Therefore, we considered that studies conducted solely in clinical settings were insufficient to identify associations between autistic traits and antenatal pain among pregnant women. Specifically, we focused on the association between autistic traits and antenatal pain among pregnant women from the general population, corresponding to a real-world scenario.

We aimed to investigate whether the presence of autistic traits among pregnant women was positively associated with antenatal pain and clarify the interaction of psychological risk factors in the association between autistic traits and antenatal pain using a large dataset from the Japanese national birth cohort study. In addition, because several clinical cases have reported that individuals with extreme autistic traits expressed extraordinary amplified pain that seriously interfered with social activity^24,25^, we examined the association between maternal autistic traits and severity of antenatal pain.

## Methods

### Study design

We used data from the Japan Environment and Children’s Study (JECS), which was a government-funded birth cohort study. Pregnant women were recruited for the JECS from 15 Regional Centers in Japan from January 2011 to March 2014. The JECS protocol has been published elsewhere^26,27^. Two self-reported questionnaires were administered during the first trimester of pregnancy (Survey 1) and again in the second to third trimester (Survey 2). The present study was based on the JECS dataset released in June 2016 and revised in October 2016 (jecs-ag-20160424).

### Study population

**Figure 1:**
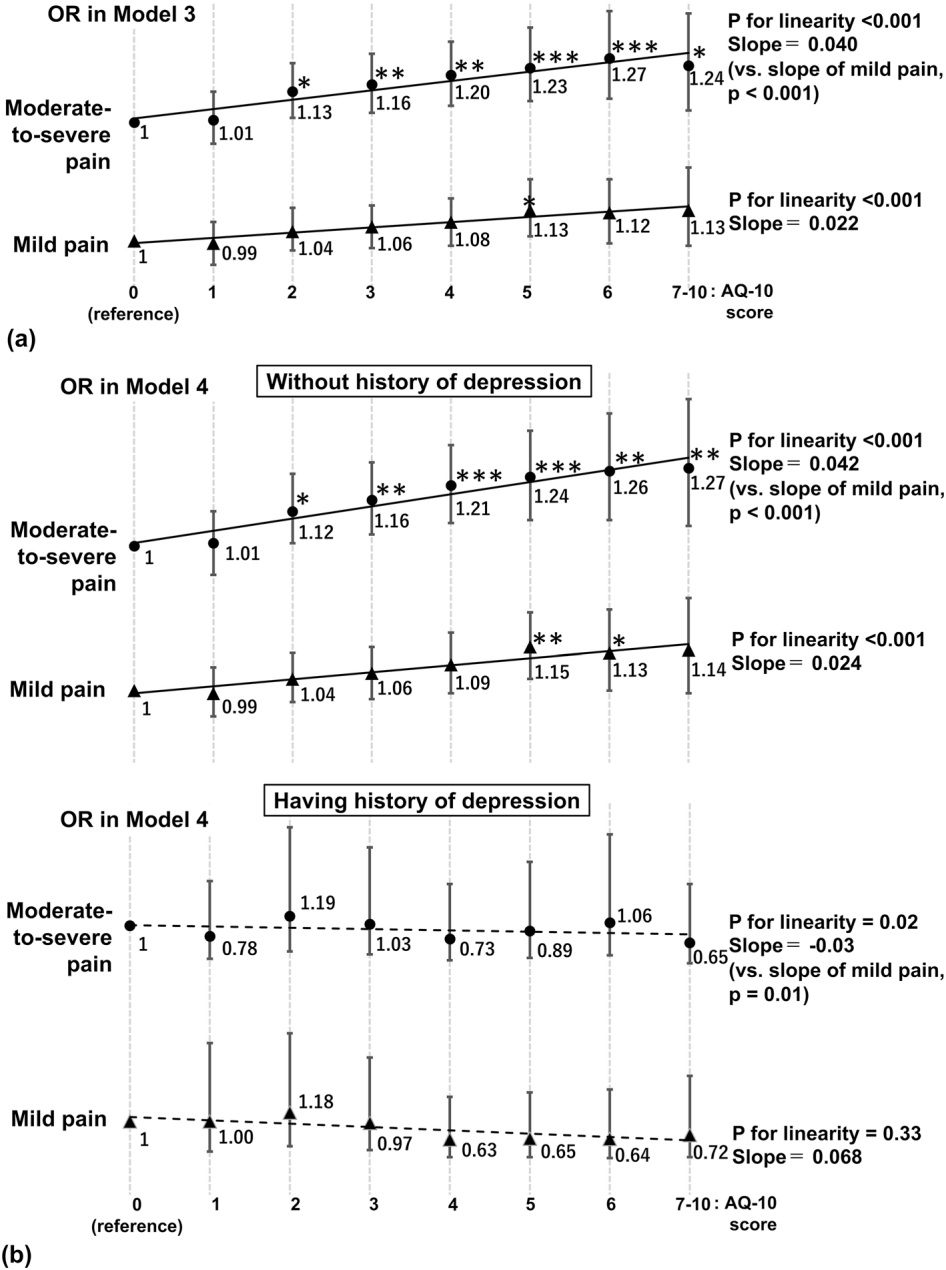
Enrollment process. Expectant mothers in the first trimester were recruited from January 2011 to March 2014. Two surveys were conducted; the first survey was conducted during the first trimester of pregnancy, and the second survey during the second to third trimester. *AQ-10-J* Autism-Spectrum Quotient short form-Japanese version.

The participant enrolment process is shown in Fig. 1. A total of 103,099 pregnancies were registered. We excluded 5645 women that were registered two and three times. We also excluded 15 women who were aged under 16 years because the original Autism Quotient used in the present study was designed for individuals aged ≥ 16 years^28^, and 3281 women with incomplete data on age at registration. In addition, we excluded 459 women with history of rheumatoid disease, ulcerous colitis, or Crohn’s disease; these pain-related diseases were considered potential confounders and the number of cases was too small to adjust for these confounders statistically. Moreover, we excluded a further 4646 women with incomplete data for pain (measured using the SF-8 bodily pain question; the SF-8-Pain) and autistic traits (measured using the Japanese version of the Autism-Spectrum Quotient short form; AQ-10-J) in the second to third trimester of pregnancy. Finally, we analyzed data for 89,068 pregnant women.

### Main measures

#### Outcome variable: pain intensity during pregnancy in the second to third trimester

The SF-8-Pain was used to assess the level of pain experienced during pregnancy^29,30^. Participants were asked, “How much bodily pain have you had during the past 4 weeks?” Response were on a six-point scale: none (1), very mild (2), mild (3), moderate (4), severe (5), and very severe (6)^29,30^. Based on these results, we categorized participants into three groups: “without pain” (responses of 1), “mild pain” (responses of 2 or 3), and “moderate-to-severe pain” (responses of 4, 5, or 6).

In this study, we used pain assessment data that was collected in Survey 2 (second to third trimester). The JECS measured maternal autistic traits once using a questionnaire in Survey 2, which was described as an experimental variable. Although autistic traits are congenital and scores for the questionnaire covering adult autistic traits were theoretically stable, we selected data for measures of maternal autistic traits and pain from the same time to avoid potential bias from temporal change in questionnaire scores.

#### Experimental variable: autistic traits

In Survey 2, autistic traits were assessed using the AQ-10-J, which is a short form of the Autism-Spectrum Quotient (AQ) and widely used in general populations^28,31^. The AQ-10-J used in the present study includes 10 items^31^: Item 1. I prefer to do things with others rather than on my own; Item 2. Other people frequently tell me that what I’ve said is impolite, even though I think it is polite; Item 3. I tend to have very strong interests, which I get upset about if I can’t pursue; Item 4. When I’m reading a story, I find it difficult to work out the character’s intentions; Item 5. I would rather go to the theater than to a museum; Item 6. I am often the last to understand the point of a joke; Item 7. I find it easy to work out what someone is thinking or feeling just by looking at their face; Item 8. I like to collect information about categories of things (e.g., types of cars, birds, trains, plants); Item 9. I find it difficult to imagine what it would be like to be someone else; and Item 10. I find it difficult to work out people’s intentions. Responses are scored using a binary system, whereby endorsement of the autistic trait (either mildly or strongly) is scored as + 1, whereas the opposite response is scored as a 0. This means that the maximum AQ-10-J score is 10. That is, for Items 1 and 7, responses of “definitely agree or slightly agree” were scored as 0, and responses of “slightly disagree or definitely disagree” as + 1. For Items 2–6 and 8–10, responses of “definitely agree or slightly agree” were scored as + 1, and responses of “slightly disagree or definitely disagree” as 0. A previous study identified an AQ-10-J score of 7 as the cut-off point to indicate probable high-functioning pervasive developmental disorders as defined by the Diagnostic and Statistical Manual of Mental Disorders, Fourth Edition (DSM-IV). This was categorized as ASD in the fifth edition of the DSM (DSM-5)^32^.

We divided pregnant women into eight scoring groups: seven consecutive scoring groups from 0 to 6, with the final group comprising those above the cut-off for ASD (AQ-10-J score ≥ 7). Because the number of women with a high AQ-10-J score was too small to obtain appropriate estimation by multivariate analyses as used in the present study and considering the clinical implications, we categorized participants above the cut-off for probable high-functioning ASD into the same scoring group.

We also divided pregnant women into five groups for the first sensitivity analysis (#1): those that fell in quartiles below the cut-off (AQ-10-J quartiles 1 [low] to 4 [high]) and above the cut-off (AQ-10-J cut-off). This categorization was adapted from a previous study^33,34^.

The Cronbach’s alpha for the AQ-10-J was 0.51 in the present study, which indicated moderate reliability^35^. In a previous validation study, the Cronbach’s alpha for the AQ-10-J was 0.61 among 25 normally intelligent patients with pervasive developmental disorders and 215 healthy individuals^31^.

### Potential confounders

#### Demographic factors

The demographic factors included as potential confounders were age at study entry (16–19, 20–24, 25–29, 30–34, 35–39, or ≥ 40 years), body mass index during non-pregnancy (quintile), smoking status during pregnancy (never smoked, former smoker, or current smoker) at Survey 2, drinking status during pregnancy (never drank, former drinker, or drinker) at Survey 2, and physical activity during pregnancy (quintile, metabolic equivalent-hour per day) at Survey 2.

Physical activity was measured using the International Physical Activity Questionnaire^36,37^.

#### Socioeconomic factors

The socioeconomic factors included as confounders were education level (below high school, high school, college/vocational school, university, or graduate school) at Survey 2, equivalized income (quintile, Japanese yen) at Survey 2, marital status (married or common-habits, single, or divorced/widowed) at Survey 1, and current employment status at Survey 2 (permanent full-time employee, self-employed, temporary full-time employee, full-time homemaker or leave of absence, part-time employee, unemployed, or others).

Equivalized income was calculated by dividing the median value of the multiple-choice response for annual household income by the square root of the number of people living in the household; number of family members (0, 1, 2, or ≥ 3) at Survey 1. Based on the poverty line in Japan in 2015^38^, a low income was defined as an annual equivalized income of less than 1.22 million Japanese yen.

#### Medical history and psychological risk factors

We collected the number of fetuses (singleton or multiple pregnancy) based on participants’ medical records. We also collected data related to participant-reported medical history and psychological risk factors, including: history of anxiety disorder (yes/no), history of depression (yes/no), history of schizophrenia (yes/no), history of other psychological disorders (yes/no), history of delivery (yes/no) at Survey 1; feeling when made aware of the pregnancy (very happy, unintended pregnancy but felt happy, unintended pregnancy and confused/upset, or no specific feeling/other feeling), psychological distress during pregnancy (yes/no) at Survey 1; and depth of sleep over the past month (quite lightly, lightly, normal, deeply, or quite deeply) at Survey 2.

Kessler Psychological Distress Scale (K6) scores ≥ 13 at Survey 2 were interpreted as indicating the presence of psychological distress^39^. We also included participant-reported history of developmental disorder (autism, Asperger’s syndrome, or pervasive developmental disorders) (yes/no); the criteria used in the self-reported questionnaire were based on the DSM-IV.

### Statistical analyses

First, we compared participant characteristics between women below and above the AQ-10-J cut-off point using t-tests for continuous variables and chi-square analyses for categorical variables. Next, we examined the association between maternal autistic traits and the prevalence of antenatal pain (mild and moderate-to-severe pain). Odds ratios (OR) for the prevalence of mild and moderate-to-severe pain for the AQ-10-J scoring groups were calculated using a multinominal logistic regression analysis adjusted for potential confounders. We used a multivariate multinominal logistic regression analysis with a generalized logit model that had two intercepts and two slopes to assess whether autistic traits were positively associated with antenatal pain, and whether autistic traits were more strongly associated with moderate-to-severe pain compared with mild pain. A general linear model was used to estimate P for linearity for the ORs for mild and moderate-to-severe pain (by AQ-10-J scoring group). Regression coefficients for the ORs for the AQ-10-J scoring groups (i.e., slopes) between mild pain and moderate-to-severe pain were also tested using a general linear model. The outcome variables (mild pain and moderate-to-severe pain) were ordinal. Although proportional odds ordinal logistic regression analysis using a generalized logit model that had different intercepts, but the same slope is often used to assess ordinal outcomes, this analysis was not appropriate for comparing the strengths of the associations in this study.

In addition, we tested whether there were any interactions of psychological risk factors in the association between AQ-10-J score and antenatal pain. The psychological risk factors considered were confusion/upset when made aware of the pregnancy, history of anxiety disorder, history of depression, history of schizophrenia, history of other psychological disorders, sleeping quite lightly, and psychological distress during pregnancy.

Based on these tests for the interactions of psychological risk factors, we stratified all pregnant women by the significant factor (i.e., history of depression) to determine the associations between AQ-10-J scoring groups and antenatal pain.

Model 1 was adjusted for age. Model 2 added pre-pregnancy body mass index, smoking during pregnancy, drinking during pregnancy, physical activity, education, marital status, equivalized income, employment status, and multiple pregnancy. Model 3 further added history of delivery, history of anxiety disorder, history of depression, history of schizophrenia, history of other psychological disorders, feeling when made aware of the pregnancy, self-reported sleep depth, and psychological distress during pregnancy. Missing values for potential confounders were used as dummy variables. Model 4 was adjusted for all variables in Model 3, except for history of depression.

For the second sensitivity analysis (#2), we re-ran a multivariate multinomial logistic regression analysis for 71,206 pregnancies without psychological risk factors other than histories of developmental disorders (autism, Asperger’s syndrome, or pervasive developmental disorder). All statistical analyses were conducted using SAS, Version 9.4 (SAS Institute Inc., Cary, NC, USA). P-values < 0.05 (two-tailed tests) were considered statistically significant.

### Ethical issues

All procedures were in accordance with the ethical standards of the Helsinki Declaration of 1975, as revised in 2010. The JECS protocol was approved by the Ministry of the Environment’s Institutional Review Board on Epidemiological Studies, and by the ethics committees at all participating institutions. Written informed consent was obtained from all participants. Authors pre-registered our hypothesis and analytic plan to the JECS Program Office, National Institute for Environmental Studies.

## Results

Table 1 shows the prevalence of antenatal pain. The prevalence of mild pain in the second to third trimester of pregnancy was 61.9%, and that of moderate-to-severe pain was 22.3%. Table 1 also shows participants’ characteristics by AQ-10-J cut-off group (i.e., presence/absence of probable ASD), with detailed information shown in Table S1. Of the 89,068 pregnant women in the study population, 20 (0.02%) women had a self-reported history of autism, Asperger’s syndrome, or pervasive developmental disorders as defined by DSM-IV. Pregnant women with history of autism, Asperger’s syndrome, or pervasive developmental disorders were also likely to be the above of the AQ-10-J cut-off for probable ASD (e.g., pervasive developmental disorders).

**Table 1:** Participants’ characteristics by the Autism-Spectrum Quotient short form (Japanese version) cut-off point (N = 89,068).

	Total		< AQ-10 cut-off		≥ AQ-10 cut-off		
			(0–6 points)		(7–10 points)		
	n = 89,068		n = 86,667		n = 2401		p-value
	n	%	n	%	n	%	< 0.001
Antenatal pain							
None	14,057	15.8	13,720	15.8	337	14.0	
Mild pain	55,121	61.9	53,657	61.9	1464	61.0	
Moderate-to-severe pain	19,890	22.3	19,290	22.3	600	25.0	
History of autism, Asperger’s syndrome, or PDD	20	0.02	9	0.01	11	0.5	< 0.001

	Mean	SD	Mean	SD	Mean	SD	
Age (years)	30.8	5.0	30.8	5.0	30.2	5.2	< 0.001

	n	%	n	%	n	%	
Pre-pregnancy overweight	9336	10.5	9092	10.5	244	10.2	0.60
Smoker during pregnancy	3948	4.4	3826	4.4	122	5.1	0.12
Drinker during pregnancy	41,774	46.9	40,716	47.0	1058	44.1	0.005
Low amounts of physical activity	19,940	22.4	19,330	22.3	610	25.4	< 0.001
Less than high school education	4184	4.7	4028	4.6	156	6.5	< 0.001
Divorced or widowed	763	0.9	732	0.8	31	1.3	0.02
Low income	29,762	33.4	28,875	33.3	887	36.9	< 0.001
Full-time homemaker	39,424	44.3	38,281	44.2	1,143	47.6	< 0.001
Multiple pregnancy	867	1.0	844	1.0	23	1.0	0.94
History of delivery	43,656	49.0	42,608	49.2	1048	43.6	< 0.001
History of anxiety disorder	2531	2.8	2397	2.8	134	5.6	< 0.001
History of depression	2703	3.0	2557	3.0	146	6.1	< 0.001
History of schizophrenia	151	0.2	139	0.2	12	0.5	< 0.001
History of other psychological disorders	871	1.0	827	1.0	44	1.8	< 0.001
Confusion/upset when made aware of the pregnancy	6592	7.4	6332	7.3	260	10.8	< 0.001
Sleeping quite lightly	6378	7.2	6147	7.1	231	9.6	< 0.001
Psychological distress during pregnancy	2914	3.3	2699	3.1	215	9.0	< 0.001

*AQ-10* Autism-Spectrum Quotient short form, *PDD* pervasive developmental disorders, *SD* standard deviation.

Criteria for developmental disorders in the questionnaire were based on the Diagnostic and Statistical Manual of Mental Disorders, Fourth Edition (DSM-IV). Body mass index ≥ 25 kg/m^2^ was defined as overweight. The first quintile of amounts of physical activity was defined as low amounts of physical activity. Equivalized income < 1.22 million Japanese yen was defined as low income. A Kessler Psychological Distress Scale (K6) score ≥ 13 was interpreted as indicating the presence of psychological distress during pregnancy. T-tests were used to compare groups on continuous variables. Chi-square analyses were conducted for categorical data.

Compared with women below the AQ-10-J cut-off, women above the AQ-10-J cut-off were more likely to be younger, have smoked during pregnancy, be divorced or widowed, have a low income, be full-time homemaker, have a history of psychological disorders, feel confused or upset when made aware of their pregnancy, sleep quite lightly, and have psychological distress during pregnancy. These women were also less likely to be alcohol drinkers during pregnancy, be physically active, have graduated from high school, and have a history of delivery compared with women below the AQ-10-J cut-off.

**Figure 2:**
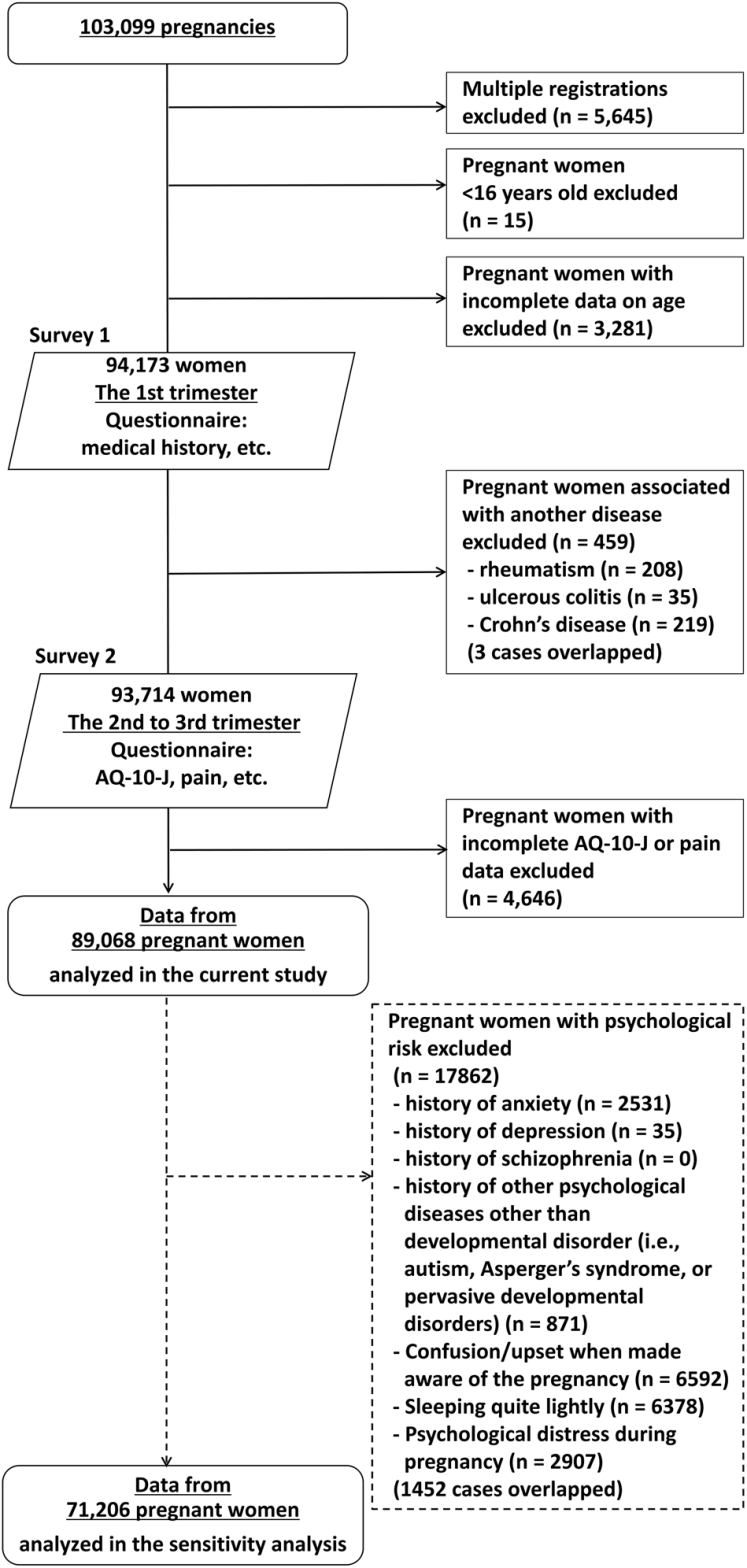
(**a**) Odds ratios for antenatal pain in Model 3; (**b**) odds ratios for antenatal pain stratified by the presence of a history of depression in Model 4. The X axis indicates AQ-10-J scoring group and the Y axis the ORs for mild and moderate-to-severe pain. The bars indicate 95% confidence intervals. P for linearity for the ORs for mild and moderate-to-severe pain by increased AQ-10-J scores were estimated using a general linear model. Regression coefficients for the ORs for the AQ-10-J scoring groups (i.e., slopes) between mild pain and moderate-to-severe pain were also tested using a general linear model. *AQ-10* Autism-Spectrum Quotient short form-Japanese version, *OR* odds ratio.

Table 2 shows the association between maternal autistic traits and the prevalence of antenatal pain. In Models 1 and 2, maternal autistic traits were positively associated with mild and moderate-to-severe pain in a dose–response manner, whereas the ORs for mild pain in the AQ-10-J scoring groups of 1–3 were not statistically significant compared with the 0 scoring group. Further adjustment (Model 3) attenuated these associations and showed that maternal autistic traits were nearly positively associated with moderate-to-severe pain in a dose–response manner (Fig. 2a), whereas most ORs for mild pain were not statistically significant. Maternal autistic traits were more strongly associated with moderate-to-severe pain than with mild pain (the slope of the ORs for moderate-to-severe pain was less than that for mild pain; p = 0.001 in Model 1, and p < 0.001 in Models 2 and 3).

**Table 2:** Odds ratios (95% confidence intervals) for antenatal pain by Autism-Spectrum Quotient short form (Japanese version) scoring group.

AQ-10-J score	Model 1		Model 2		Model 3	
	Mild pain	Moderate-to-severe pain	Mild pain	Moderate-to-severe pain	Mild pain	Moderate-to-severe pain
0: n = 4931	3108	979	3108	979	3108	979
	1 (reference)	1 (reference)	1 (reference)	1 (reference)	1 (reference)	1 (reference)
1: n = 15,176	9469	3120	9469	3120	9469	3120
OR (95% CI)	1.00 (0.91–1.09)	1.04 (0.94–1.16)	1.00 (0.91–1.09)	1.03 (0.93–1.15)	0.99 (0.90–1.08)	1.01 (0.91–1.13)
2: n = 22,659	14,063	4975	14,063	4975	14,063	4975
OR (95% CI)	1.06 (0.97–1.15)	1.19 (1.07–1.31)***	1.07 (0.97–1.15)	1.17 (1.06–1.30)**	1.04 (0.96–1.14)	1.13 (1.02–1.25)*
3: n = 20,410	12,601	4615	12,601	4615	12,601	4615
OR (95% CI)	1.07 (0.99–1.17)	1.25 (1.13–1.38)***	1.08 (0.99–1.17)	1.22 (1.10–1.36)***	1.06 (0.97–1.15)	1.16 (1.04–1.29)**
4: n = 12,550	7698	2955	7698	2955	7698	2955
OR (95% CI)	1.11 (1.01–1.21)*	1.34 (1.20–1.49)***	1.11 (1.01–1.22)*	1.30 (1.17–1.45)***	1.08 (0.98–1.18)	1.20 (1.07–1.34)**
5: n = 7218	4457	1729	4457	1729	4457	1729
OR (95% CI)	1.16 (1.05–1.29)**	1.41 (1.25–1.59)***	1.17 (1.06–1.30)**	1.38 (1.22–1.55)***	1.13 (1.02–1.26)*	1.23 (1.09–1.40)***
6: n = 3723	2261	926	2261	926	2261	926
OR (95% CI)	1.15 (1.02–1.30)*	1.47 (1.28–1.69)***	1.16 (1.03–1.31)*	1.44 (1.25–1.66)***	1.12 (0.99–1.26)	1.27 (1.10–1.47)***
7–10: n = 2401	1464	600	1464	600	1464	600
OR (95% CI)	1.18 (1.03–1.36)*	1.51 (1.29–1.78)***	1.20 (1.04–1.38)*	1.49 (1.27–1.75)***	1.13 (0.98–1.31)	1.24 (1.05–1.46)*
P for linearity	< 0.001	< 0.001	< 0.001	< 0.001	< 0.001	< 0.001
Regression coefficient (Slope) for ORs of the AQ-10-J scoring groups	0.028	0.077 (vs. mild pain, p = 0.001)	0.030	0.074 (vs. mild pain, p < 0.001)	0.022	0.040 (vs. mild pain, p < 0.001)
Potential confounders						
History of anxiety disorder, No: reference, Yes: OR (95% CI)	–	–	–	–	1.53 (1.32–1.78)***	1.93 (1.65–2.26)***
History of depression, No: reference, Yes: OR (95% CI)	–	–	–	–	1.12 (0.98–1.28)	1.62 (1.41–1.87)***
History of schizophrenia, No: reference, Yes: OR (95% CI)					1.17 (0.67–2.04)	1.26 (0.69–2.30)***
History of other psychological disorders, No: reference, Yes: OR (95% CI)					1.14 (0.91–1.42)	1.64 (1.29–2.08)***
Feeling when made aware of the pregnancy						
Very happy	–	–	–	–	(reference)	(reference)
Unintended pregnancy but felt happy OR (95% CI)	–	–	–	–	1.14 (1.09–1.20)***	1.19 (1.13–1.26)***
Unintended pregnancy and confused/upset OR (95% CI)	–	–	–	–	1.19 (1.13–1.28)***	1.45 (1.33–1.59)***
No specific feeling/other feeling OR (95% CI)	–	–	–	–	1.12 (0.95–1.32)	1.45 (1.21–1.74)***
Depth of sleep in the past month						
Sleeping quite deeply	–	–	–	–	(reference)	(reference)
Sleeping deeply OR (95% CI)	–	–	–	–	1.40 (1.23–1.59)***	1.22 (1.03–1.44)*
Normal OR (95% CI)	–	–	–	–	1.60 (1.42–1.81)***	1.43 (1.23–1.67)***
Sleeping lightly OR (95% CI)	–	–	–	–	2.33 (2.06–2.63)***	2.88 (2.47–3.37)***
Sleeping quite lightly OR (95% CI)	–	–	–	–	2.61 (2.25–3.02)***	5.08 (4.26–6.07)***
Psychological distress, No: reference, Yes: OR (95% CI)	–	–	–	–	1.64 (1.40–1.92)***	3.36 (2.87–3.94)***

*AQ-10-J* Japanese version of the Autism-Spectrum Quotient short form, *CI* confidence interval, *OR* odds ratio.

Model 1: Adjusted for age.

Model 2: Adjusted for age, pre-pregnancy body mass index, smoking during pregnancy, drinking during pregnancy, physical activity, education, marital status, equivalized income, employment status, and multiple pregnancy.

Model 3: Further adjusted for history of delivery, history of anxiety disorder, history of depression, history of schizophrenia, history of other psychological disorders, feeling when made aware of the pregnancy, sleep depth, and psychological distress during pregnancy.

The Kessler Psychological Distress Scale (K6) score ≥ 13 was interpreted as indicating the presence of psychological distress during pregnancy.

Odds ratio was estimated by multinominal logistic regression analysis. p for linearity for the ORs for mild and moderate-to-severe pain by increase in AQ-10-J score were estimated using a general linear model. Regression coefficients for the ORs in the AQ-10-J scoring groups (i.e., slopes) between mild pain and moderate-to-severe pain were also tested using a general linear model. *p < 0.05, **p < 0.01, ***p < 0.001.

Analysis of potential confounders showed unintended pregnancy and psychological distress were independently associated with a higher prevalence of mild and moderate-to-severe pain compared with felling happy when made aware of the pregnancy and no psychological distress. Sleeping deeply, normally, lightly, and quite lightly in the past month were also independently associated with a higher prevalence of mild and moderate-to-severe pain compared with sleeping quite deeply. Feeling “nothing at all” and “other feelings” when made aware of the pregnancy were associated with a higher prevalence of moderate-to-severe pain, but not with a higher prevalence of mild pain.

Among the interactions of psychological risk factors examined, we found that the presence of a history of depression had a significant interaction in the association between AQ-10-J scoring group and antenatal pain.

Table 3 and Fig. 2b show the association between maternal autistic traits and the prevalence of antenatal pain stratified by the presence of a history of depression. The results for the association between maternal autistic traits and antenatal pain among pregnant women without a history of depression were similar to those for all pregnant women. However, in pregnant women with a history of depression, there was no association between maternal autistic traits and antenatal pain.

**Table 3:** Odds ratios (95% confidence intervals) for antenatal pain by Autism-Spectrum Quotient short form (Japanese version) scoring group: stratification by the presence of a history of depression.

	Model 4	
Without a history of depression, n = 86,365	Mild pain	Moderate-to-severe pain
AQ-10-J score		
0: n = 4885	3063	955
OR (95% CI)	1 (reference)	1 (reference)
1: n = 14,873	9311	3006
OR (95% CI)	0.99 (0.91–1.08)	1.01 (0.90–1.12)
2: n = 22,062	13,717	4773
OR (95% CI)	1.04 (0.96–1.13)	1.12 (1.01–1.25)*
3: n = 19,807	12,262	4407
OR (95% CI)	1.06 (0.97–1.15)	1.16 (1.04–1.29)**
4: n = 12,090	7463	2791
OR (95% CI)	1.09 (0.99–1.20)	1.21 (1.08–1.35)***
5: n = 6905	4605	1596
OR (95% CI)	1.15 (1.04–1.27)**	1.24 (1.09–1.40)***
6: n = 3518	2168	836
OR (95% CI)	1.13 (1.00–1.28)*	1.26 (1.09–1.46)**
7–10: n = 2255	1382	552
OR (95% CI)	1.14 (0.99–1.32)	1.27 (1.07–1.51)**
P for linearity	< 0.001	< 0.001
Regression coefficient (the slope) for ORs of AQ-10-J scoring groups	0.024	0.042 (vs. mild pain, p < 0.001)

With a history of depression, n = 2703	Mild pain	Moderate-to-severe pain
AQ-10-J score		
0: n = 76	45	24
OR (95% CI)	1 (reference)	1 (reference)
1: n = 303	158	114
OR (95% CI)	1.00 (0.38–2.60)	0.78 (0.32–1.91)
2: n = 597	346	202
OR (95% CI)	1.18 (0.49–2.80)	1.19 (0.47–3.01)
3: n = 603	339	208
OR (95% CI)	0.97 (0.41–2.30)	1.03 (0.41–2.60)
4: n = 460	235	164
OR (95% CI)	0.63 (0.27–1.50)	0.73 (0.29–1.85)
5: n = 313	152	124
OR (95% CI)	0.65 (0.27–1.59)	0.89 (0.34–2.30)
6: n = 205	93	90
OR (95% CI)	0.64 (0.25–1.65)	1.06 (0.39–2.86)
7–10: n = 146	82	48
OR (95% CI)	0.72 (0.27–1.93)	0.65 (0.23–1.85)
P for linearity	0.02	0.33
Regression coefficient (the slope) for ORs of AQ-10-J scoring groups	− 0.068	− 0.03 (vs. mild pain, p = 0.01)

*AQ-10-J* Japanese version of the Autism-Spectrum Quotient short form, *CI* confidence interval, *OR* odds ratio.

Model 4: Adjusted for age, pre-pregnancy body mass index, smoking during pregnancy, drinking during pregnancy, physical activity, education, marital status, equivalized income, employment status, multiple pregnancy. History of delivery, history of anxiety disorder, history of schizophrenia, history of other psychological disorders, feeling when pregnancy was found out, sleep depth, and psychological distress.

The Kessler Psychological Distress Scale (K6) score ≥ 13 was interpreted as indicating the presence of psychological distress during pregnancy.

Odds ratio was estimated by multinominal logistic regression analysis. P for linearity for the ORs for mild and moderate-to-severe pain by increase in AQ-10-J score were estimated using a general linear model. Regression coefficients for the ORs in the AQ-10-J scoring groups (i.e., slopes) between mild pain and moderate-to-severe pain were also tested using a general linear model. *p < 0.05, **p < 0.01, ***p < 0.001.

The sensitivity analysis #1 using different AQ-10-J scoring groups from the main analyses (Table S2) and sensitivity analysis #2 that excluded pregnancies with psychological factors other than history of developmental disorders (autism, Asperger’s syndrome, or pervasive developmental disorders) revealed similar results to the main results (Tables S3 and S4).

## Discussion

Maternal autistic traits were positively associated with antenatal mild and moderate-to-severe pain in a dose–response manner. Additionally, maternal autistic traits were more strongly associated with moderate-to-severe antenatal pain compared with mild pain. Moreover, we identified that a history of depression had an interaction in the association between maternal autistic traits and antenatal pain. Specifically, the association between autistic traits and antenatal pain was confined to pregnant women without a history of depression. To our knowledge, this is the first study to show a positive association between maternal autistic traits and antenatal pain along with several psychosocial risk factors in a sample from the general population.

A previous study used the original AQ was used to measure autistic traits, including broader autism phenotype, in the general population^40,41^. Autistic traits are clinically defined in graduations. Therefore, we considered the validated AQ-10-J was suitable for assessing autistic traits in the national birth cohort used in the present study. The proportion women with a history of ASD and the prevalence of probable ASD (i.e., above the AQ-10-J cut-off) were 0.2% and 2.7%, respectively. Few large-scale epidemiological studies have examined neurodevelopmental disorders (including autistic traits) in Japan. However, a study that investigated children/students with probable developmental disabilities who needed educational support in regular classes was conducted by Japan’s Ministry of Education, Culture, Sports Science, and Technology. That study reported that 1.8% of male students and 0.4% of female students (among 52,272 students in public elementary and junior high schools) presented with probable ASD as measured by the High-Functioning Autism Spectrum Screening Questionnaire (ASSQ)^42^. The proportion of those with a history of ASD in this study was similar to that of Japanese female students reported in the previous study, but the prevalence of probable ASD in our study was higher than in the previous study. This discrepancy may result from the differences in measurement between the ASSQ and AQ-10-J, or differences between girls and adult women. In addition, people with high-functioning ASD are often only diagnosed when in adulthood^43^.

Our results may be explained by a higher pain response among women with autistic traits compared with women without these traits. However, the underlying reason for this remains unclear. A previous study showed greater variation in pain sensitivity among individuals with ASD than those without ASD^12^. Moreover, previous research using functional magnetic resonance imaging (fMRI) reported that people with ASD had different central responses to pleasant and unpleasant stimulation than those without ASD^13^. Using three different stimulations, including those that were unpleasant (a plastic mesh material), neutral (burlap fabric), and pleasant (soft cosmetic brushes), those authors found that people with ASD tended to show diminished responses to pleasant and neutral stimulations, but exaggerated limbic responses to unpleasant stimulations^13^. Antenatal pain may be therefore recognized as an exaggerated unpleasant stimulation among women with ASD compared with women without ASD. This may be because of increased responses in the limbic system, which is responsible for the effect of negative affect and cognition of pain. As noted in previous research, it is possible that pregnant women with autistic traits experience more severe antenatal pain compared with pregnant women without autistic traits. However, in terms of short-term reactions to experimental heat stimulation, individuals with ASD showed intact early responses (0–10 s) but diminished sustained responses (18–28 s)^15^. During sustained responses, decreased signals in the posterior cingulate cortex were detected by fMRI^15^.

There is another possible neuropsychiatric mechanism between autistic traits and antenatal pain that could be assumed, as described below. Individuals with ASD have been found to have decreased gray matter in the posterior superior lobule, which suggests cognitive rigidity against external stimulations such as pain^44^. Moreover, individuals who experienced more severe pain also displayed significant impairment in attention and cognitive flexibility^45,46^. Although these potential overlaps between alterations of neural function that may contribute to autistic traits and persistent pain could be occasional, causality remains unknown, and it is possible that pregnant women with autistic traits pay sustained attention to pain.

Pain processing is related to a sensitivity (nociceptive and nocifensive) component, and to emotional-affective and cognitive components that are regulated by the interoceptive system^20,47,48^. Consistent with this mechanism, we found a higher prevalence of antenatal pain in participants with independent psychological risk factors compared with those without psychological risk factors. However, contrary to our hypothesis, the interactions of psychological risk factors other than history of depression in the association between maternal autistic traits and antenatal pain were not significant. Stratification of participants by the presence of a history of depression showed no association between autistic traits and antenatal pain in pregnant women with a history of depression. However, many patients with depression have comorbid chronic pain^49^, which might have masked an association between maternal autistic traits and antenatal pain.

Interventions for individuals with extreme autistic traits (e.g., ASD) can be feasible by cognitive-behavioral treatment for anxiety or depressive symptoms, focused social skills training, speech therapy, family education and interventions, and academic assessment to improve their difficulty in social life^25^. A previous narrative qualitative study found communication difficulties in pregnant women with ASD led to perceived stress and anxiety about childbirth, which suggested that women with communication challenges need special attention^21^. For example, recommendations included using written information for explanations or other methods of instruction (e.g., online resources) and frequent confirmation of understanding^21^. However, an observational study with a cross-sectional design could not conclude whether these interventions reduced pain intensity during pregnancy through improving symptoms related to maternal autistic traits. Further research such as randomized controlled trials is required to investigate the effect of these interventions on pain symptoms among pregnant women. However, the findings of the present study provided evidence that may inform changes in the treatment of antenatal pain in this population.

Pregnant women with moderate-to-severe pain may have comorbid extreme autistic traits. The present study highlighted the importance of screening for autistic traits among pregnant women in the treatment and control of severe antenatal pain. If pregnant women with severe pain have comorbid extreme autistic traits, they may benefit from interventions to improve their overall functioning and decrease pain and pain-associated disability. The prevalence of ASD among women is lower than that among men^50^, and women may be less likely to be suspected to have and diagnosed with autistic traits than men. Comorbid autistic traits should therefore be considered when healthcare providers encounter pregnant women with severe antenatal pain, and healthcare professionals should carefully consider optimized interventions when providing care for pregnant women with autistic traits.

The strength of the present study was that our data were derived from a large nation-wide cohort study of the general population. However, there are several limitations to consider when interpreting the findings of this study. First, we did not examine the site of pain and therefore could not distinguish brief pain such as possible headache, lower back pain, or pelvic pain. In addition, we did not have information about whether antenatal pain started during or before pregnancy. This information may have clinical implications. Second, both the experimental variable (AQ-10-J) and the outcome variable (pain intensity) comprised a small number of items, which might have reduced the variance of responses and generalizability of the results. However, many questionnaires were required for the multidimensional assessment of nearly 10,000 participants in the JCES. As such, it was important to reduce burden on responders by using brief questionnaires where possible. Third, ASD and ADHD traits were highly coexistence in a general adult population^51^. The autistic traits also overlap with psychological distress such as depression^52^. Therefore, the AQ-10-J responded not only to individuals with autistic traits but also to those with ADHD traits and psychological distress such as depression^51,52^. The AQ was indeed correlated with the Adult ADHD Self-Report Scale (ASRS) in the general population^52^. This situation may influence the interpretation of the results. Fourth, this study used a cross-sectional design, and temporal aspects cannot be discussed. However, autistic traits are innate, and it may not be necessary to consider the possibility of reverse causality (i.e., whether antenatal pain caused maternal autistic traits).Fifth, the associations that we observed could be explained by potential uncontrolled confounders. These confounders may include factors that occurred before or during the antenatal period, such as genetic predisposition and exposure to other environmental factors. Sixth, the validation study of the AQ-10-J^31^ showed a lower positive predictive value than the previous study^53^ despite having similar findings of sensitivity and specificity among them. This insufficient psychometric property might cause misclassification; those with high AQ-10-J score might not be that autistic, resulting in an overestimation of the association between maternal autistic traits and antenatal pain.

In conclusions, maternal autistic traits are positively associated with antenatal pain in a dose–response manner, with this association strongest for moderate-to-severe antenatal pain compared with mild pain, after adjusting for psychological confounders. Our findings suggest that maternal autistic traits may have a key role in antenatal pain and should be considered when providing healthcare for expectant mothers.

## Supplementary Information

Supplementary Information.

Supplementary Tables.

## Data Availability

Data are unsuitable for public deposition due to ethical restrictions and legal framework of Japan. It is prohibited by the Act on the Protection of Personal Information (Act No. 57 of 30 May 2003, amendment on 9 September 2015) to publicly deposit the data containing personal information. Ethical Guidelines for Medical and Health Research Involving Human Subjects enforced by the Japan Ministry of Education, Culture, Sports, Science and Technology and the Ministry of Health, Labour and Welfare also restricts the open sharing of the epidemiologic data. All inquiries about access to data should be sent to: jecs-en@nies.go.jp. The person responsible for handling enquiries sent to this e-mail address is Dr Shoji F. Nakayama, JECS Program Office, National Institute for Environmental Studies.
