# Supplementary material for: Maternal autistic traits and antenatal pain by cross-sectional analysis of the Japan Environment and Children’s Study
Source: Sci Rep. 2023 Apr 13;13:6068. doi: 10.1038/s41598-023-32945-2 (PMC10101964; doi:10.1038/s41598-023-32945-2)
Supplement: Supplementary file 2 — Supplementary Tables. [file 41598_2023_32945_MOESM2_ESM.pdf]

Table S1. Participants' characteristics by the Autism-Spectrum Quotient short form (Japanese version) cut-off of point (N = 89,068)

	**Total**		**< AQ-10 Cut-off**		**≥ AQ-10 Cut-off**		
			**0-6 points**		**7-10 points**		
	**n = 89,068**		**n = 86,667**		**n = 2,401**		P value
	**n**	**%**	**n**	**%**	**n**	**%**	
**Antenatal pain**							0.002
None	14,057	15.8	13,720	15.8	337	14.0	
Very mild	20,606	23.1	20,064	23.2	542	22.6	
Mild	34,515	38.8	33,593	38.8	922	38.4	
Moderate	15,665	17.6	15,204	17.5	461	19.2	
Severe	3,870	4.3	3,749	4.3	121	5.0	
Very severe	355	0.4	337	0.4	18	0.7	
**Age, years**							<0.001
16-19	952	1.1	910	1.0	42	1.7	
20-24	8,965	10.1	8672	10.0	293	12.2	
25-29	26,094	29.3	25350	29.2	744	31.0	
30-34	31,125	34.9	30327	35.0	798	33.2	
35-39	18,684	21.0	18237	21.0	447	18.6	
≥40	3,248	3.6	3171	3.7	77	3.2	
**Pre-pregnancy body mass index ≥25, kg/m^2**							0.68
<18.5	14,375	16.1	13970	16.1	405	16.9	
18.5 to 24.9	65,322	73.3	63570	73.3	1752	73.0	
25.0 to 29.9	7,149	8.0	6959	8.0	190	7.9	
≥30	2,187	2.5	2133	2.5	54	2.2	
Missing	35	0.04	35	0.04	0	0	
**Smoking during pregnancy**							0.07
Never smoker	51,230	57.5	49906	57.6	1324	55.1	
Ex-smoker	33,191	37.3	32258	37.2	933	38.9	
Smoker	3,948	4.4	3826	4.4	122	5.1	
Missing	699	0.8	677	0.8	22	0.9	
**Drinking during pregnancy**							0.01
Never drinker	29,517	33.1	28702	33.1	815	33.9	
Ex-drinker	56,403	63.3	54929	63.4	1474	61.4	
Drinker	2,434	2.7	2350	2.7	84	3.5	
Missing	714	0.8	686	0.8	28	1.2	

**Amounts of physical activity during pregnancy, metabolic equivalent hours per day**							0.003
Quintile 1: 0	19,940	22.4	19330	22.3	610	25.4	
Quintile 2: 0.001–0.709	14,678	16.5	14298	16.5	380	15.8	
Quintile 3: 0.710–1.715	16,406	18.4	15964	18.4	442	18.4	
Quintile 4: 1.716–4.717	17,283	19.4	16854	19.4	429	17.9	
Quintile 5: 4.718–729.943	16,660	18.7	16245	18.7	415	17.3	
Missing	4,101	4.6	3976	4.6	125	5.2	
**Education**							
Less than high school	4,184	4.7	4028	4.6	156	6.5	
High school	27,711	31.1	26827	31.0	884	36.8	
College/vocational school	37,376	42.0	36507	42.1	869	36.2	
University	18,132	20.4	17703	20.4	429	17.9	
Graduate school	1,309	1.5	1263	1.5	46	1.9	
Missing	356	0.4	339	0.4	17	0.7	
**Marital status**							<0.001
Married or common-habits	84,721	95.1	82497	95.2	2224	92.6	
Single	3,202	3.6	3072	3.5	130	5.4	
Divorced	751	0.8	720	0.8	31	1.3	
Widowed	12	0.0	12	0.0	0	0.0	
Missing	382	0.4	366	0.4	16	0.7	
**Equivalized income, million Japanese yen**							<0.001
Quintile 1: 0.19–1.14	14,050	15.8	13605	15.7	445	18.5	
Quintile 2: 1.15–1.86	19,025	21.4	18544	21.4	570	23.7	
Quintile 3: 1.87–2.99	18,538	20.8	18091	20.9	447	18.6	
Quintile 4: 3.00–4.03	13,266	14.9	12917	14.9	349	14.5	
Quintile 5: 4.04–1.77	17,800	20.0	17407	20.1	393	16.4	
Missing	6,389	7.2	6192	7.1	197	8.2	
**Current employment status**							<0.001
Permanent full-time employee	27,323	30.7	26644	30.7	679	28.3	
Self-employed	3,089	3.5	3018	3.5	71	3.0	
Temporary full-time employee	1,152	1.3	1122	1.3	30	1.2	
Full-time homemaker or leave of absence	39,424	44.3	38281	44.2	1143	47.6	
Part-time employee	14,466	16.2	14101	16.3	365	15.2	
Unemployed	1,236	1.4	1187	1.4	49	2.0	
Others	1,683	1.9	1644	1.9	39	1.6	
Missing	695	0.8	670	0.8	25	1.0	
**Number of fetuses**							0.94
Singleton pregnancy	867	1.0	844	1.0	23	1.0	

Multiple pregnancy	88,201	99.0	85823	99.0	2378	99.0	
**History of delivery**							<0.001
Yes	43,656	49.0	42608	49.2	1048	43.6	
No	45,408	51.0	44055	50.8	1353	56.4	
Missing	4	0.004	4	0.005	0	0	
**History of anxiety disorder**							<0.001
Yes	2,531	2.8	2397	2.8	134	5.6	
No	86,537	97.2	84270	97.2	2267	94.4	
**History of depression**							<0.001
Yes	2,703	3.0	2557	3.0	146	6.1	
No	86,365	97.0	84110	97.0	2255	93.9	
**History of schizophrenia**							<0.001
Yes	151	0.2	139	0.2	12	0.5	
No	88,917	99.8	86528	99.8	2389	99.5	
**History of other psychological disorders**							<0.001
Yes	871	1.0	827	1.0	44	1.8	
No	88,197	99.0	85840	99.0	2357	98.2	
**Feeling when made aware of the pregnancy**							<0.001
Very happy	58,346	65.5	56937	65.7	1409	58.7	
Unintended pregnancy but felt happy	22,414	25.2	21762	25.1	652	27.2	
Unintended pregnancy and confused	6,090	6.8	5863	6.8	227	9.5	
Upset	502	0.6	469	0.5	33	1.4	
No specific feeling	413	0.5	392	0.5	21	0.9	
Other feeling	947	1.1	905	1.0	42	1.7	
Missing	356	0.4	339	0.4	17	0.7	
**Depth of sleep over the past month**							<0.001
Quite lightly	6,378	7.2	6147	7.1	231	9.6	
Lightly	37,301	41.9	36302	41.9	999	41.6	
Normal	35,309	39.6	34406	39.7	903	37.6	
Deeply	8,241	9.3	8036	9.3	205	8.5	
Quite deeply	1,581	1.8	1530	1.8	51	2.1	
Missing	258	0.3	246	0.3	12	0.5	
**Psychological distress during pregnancy**							<0.001
Yes	2,907	3.3	2692	3.1	215	9.0	
No	85,638	96.1	83470	96.3	2168	90.3	
Missing	523	0.6	505	0.6	18	0.7	

Abbreviation: AQ-10; Autism-Spectrum Quotient short form. The Kessler Psychological Distress Scale (K6) score $\geq$13 was interpreted as indicating the presence of psychological distress during pregnancy. Chi-square analyses

were conducted for categorical data.

Table S2. Odds ratios (95% confidence intervals) of antenatal pain by quartiles of the Autism-Spectrum Quotient sort form (Japanese version) score (N = 89,068)

	**Model 1**		**Model 2**		**Model 3**	
	**Mild pain**	**Moderate-to-severe pain**	**Mild pain**	**Moderate-to-severe pain**	**Mild pain**	**Moderate-to-severe pain**
**Quartiles of AQ-10-J score**						
**1 (low): n=20,107**	**12,577**	**4,099**	14,063	4,975		
**OR (95% CI)**	1 (reference)	1 (reference)	1 (reference)	1 (reference)	1 (reference)	1 (reference)
**2: n=22,659**	14,063	4,975	14,063	4,975	14,063	4,975
**OR (95% CI)**	1.06 (1.01–1.12)*	1.15 (1.08–1.23)***	1.06 (1.01–1.12)*	1.14 (1.07–1.22)***	1.06 (1.00–1.11)*	1.12 (1.05–1.19)***
**3: n=20,410**	12,601	4,615	12,601	4,615	12,601	4,615
**OR (95% CI)**	1.08 (1.02–1.14)**	1.21 (1.13–1.29)***	1.08 (1.02–1.14)**	1.20 (1.12–1.27)***	1.07 (1.01–1.13)*	1.15 (1.08–1.23)***
**4 (high): n=23,491**	14,416	5,601	14,416	5,601	14,416	5,601
**OR (95% CI)**	1.13 (1.08–1.20)***	1.34 (1.26–1.42)***	1.14 (1.08–1.20)***	1.31 (1.23–1.40)***	1.11 (1.05–1.17)***	1.21 (1.14–1.29)***
**≥cut-off: n=2,401**	1,464	600	1,464	600	1,464	600
**OR (95% CI)**	1.19 (1.05–1.34)**	1.47 (1.28–1.69)***	1.20 (1.06–1.36)**	1.45 (1.26–1.68)***	1.15 (1.01–1.30)*	1.23 (1.06–1.42)**
**P for linearity**	<0.001	<0.001	<0.001	<0.001	<0.001	<0.001
**Regression coefficient (the slope) for ORs of AQ-10-J scoring groups**	0.045	0.113 (vs. mild pain, p=0.007)	0.048	0.107 (vs. mild pain, p=0.004)	0.035	0.055 (vs. mild pain, p=0.002)

Abbreviations: AQ; Autism-Spectrum Quotient short form, OR; odds ratio. Model 1: Adjusted for age. Model 2: Adjusted for age, pre-pregnancy body mass index, smoking during pregnancy, drinking during pregnancy, physical activity, education, marital status, equivalized income, employment status, and multiple pregnancy. Model 3: Further adjusted for history of delivery, history of anxiety disorder, history of depression, history of schizophrenia, history of other psychological disorders, feeling when made aware

of the pregnancy, sleep depth, and psychological distress during pregnancy. Odds ratio was estimated by multinominal logistic regression analysis. P for linearity for the ORs for mild and moderate-to-severe pain by increase in AQ-10-J score were estimated using a general linear model. Regression coefficients for the ORs in the AQ-10-J scoring groups (i.e., slopes) between mild pain and moderate-to-severe pain were also tested using a general linear model. * $p<0.05$, ** $p<0.01$, *** $p<0.001$

Table S3. Odds ratios (95% confidence intervals) of antenatal pain by the Autism-Spectrum Quotient sort form (Japanese version) scoring group (N = 71,206)

	Model 1		Model 2	
**AQ-10-J score**	**Mild pain**	**Moderate-to-severe pain**	**Mild pain**	**Moderate-to-severe pain**
**0: n=4,205**	2.685	766	2.685	766
**OR (95% CI)**	1 (reference)	1 (reference)	1 (reference)	1 (reference)
**1: n=12,714**	8,046	2,363	8,046	2,363
**OR (95% CI)**	0.98 (0.89–1.08)	1.01 (0.90–1.14)	0.98 (0.89–1.08)	1.01 (0.90–1.13)
**2: n=18,525**	11,714	3,646	11,714	3,646
**OR (95% CI)**	1.04 (0.96–1.14)	1.14 (1.02–1.14)*	1.05 (0.96–1.14)	1.13 (1.01–1.26)*
**3: n=16,282**	10,273	3,273	10,273	3,273
**OR (95% CI)**	1.06 (0.97–1.16)	1.18 (1.06–1.32)**	1.06 (0.97–1.16)	1.17 (1.05–1.31)**
**4: n=9,672**	6,085	1,990	6,085	1,990
**OR (95% CI)**	1.07 (0.97–1.18)	1.22 (1.08–1.38)***	1.08 (0.98–1.19)	1.21 (1.07–1.36)**
**5: n=5,425**	3,461	1,117	3,461	1,117
**OR (95% CI)**	1.15 (1.03–1.28)*	1.29 (1.13–1.47)***	1.16 (1.04–1.30)**	1.28 (1.11–1.46)***
**6: n=2,735**	1,734	576	1,734	576
**OR (95% CI)**	1.15 (1.01–1.31)*	1.32 (1.13–1.55)***	1.16 (1.02–1.33)*	1.31 (1.12–1.54)***
**7-10: n=1,648**	1,053	334	1,053	334
**OR (95% CI)**	1.13 (0.97–1.33)	1.24 (1.02–1.50)*	1.15 (0.98–1.35)	1.25 (1.03–1.51)*
**P for linearity**	<0.001	<0.001	<0.001	<0.001
**Regression coefficient (the slope) for ORs of AQ-10-J scoring groups**	0.025	0.044 (vs. mild pain, p<0.001)	0.027	0.045 (vs. mild pain, p<0.001)

Abbreviations: AQ-10-J; Japanese version of the Autism-Spectrum Quotient short form, OR; odds ratio. Model 1: Adjusted for age. Model 2: Adjusted for age, pre-pregnancy body mass index, smoking during pregnancy, drinking during pregnancy, physical activity, education, marital status, equivalized income, employment status, and multiple pregnancy. Odds ratio was estimated by multinominal logistic regression analysis. P for linearity for the ORs for mild and moderate-to-severe pain by increase in AQ-10-J score were estimated using a general linear model. Regression coefficients for the ORs in the AQ-10-J scoring groups (i.e., slopes) between mild pain and moderate-to-severe pain were also tested using a general linear model. * p<0.05, ** p<0.01, *** p<0.001

Table S4. Odds ratios (95% confidence intervals) of antenatal pain by quartiles of the Autism-Spectrum Quotient sort form (Japanese version) score (N = 71,206)

	**Model 1**		**Model 2**	
	**Mild pain**	**Moderate-to-severe pain**	**Mild pain**	**Moderate-to-severe pain**
**Quartiles of AQ-10-J score**				
**1: n=16,919**	10,731	3,129	10,731	3,129
**OR (95% CI)**	1 (reference)	1 (reference)	1 (reference)	1 (reference)
**2: n=18,525**	11,714	3,646	11,714	3,646
**OR (95% CI)**	1.06 (0.99–1.12)	1.13 (1.05–1.21)***	1.06 (1.00–1.12)	1.12 (1.05–1.21)***
**3: n=16,282**	10,273	3,273	10,273	3,273
**OR (95% CI)**	1.07 (1.01–1.14)*	1.17 (1.09–1.26)***	1.08 (1.02–1.14)*	1.17 (1.09–1.25)***
**4: n=17,832**	11,280	3,683	11,280	3,683
**OR (95% CI)**	1.12 (1.06–1.19)***	1.25 (1.16–1.34)***	1.13 (1.07–1.20)***	1.24 (1.15–1.33)***
**≥cut-off: n=1,648**	1,053	334	1,053	334
**OR (95% CI)**	1.15 (0.99–1.32)	1.23 (1.04–1.46)*	1.17 (1.02–1.35)*	1.24 (1.05–1.47)*
**P for linearity**	<0.001	<0.001	<0.001	<0.001
**Regression coefficient (the slope) for ORs of AQ-10-J scoring groups**	0.036	0.058 (vs. mild pain, p=0.003)	0.041	0.060 (vs. mild pain, p=0.014)

Abbreviations: AQ-10-J; Japanese version of Autism-Spectrum Quotient short form, OR; odds ratio. Model 1: Adjusted for age. Model 2: Adjusted for age, pre-pregnancy body mass index, smoking during pregnancy, drinking during pregnancy, physical activity, education, marital status, equivalized income, employment status, and multiple pregnancy. Odds ratio was estimated by multinominal logistic regression analysis. P for linearity for the ORs for mild and moderate-to-severe pain by increase in AQ-10-J score were estimated using a general linear model. Regression coefficients for the ORs in the AQ-10-J scoring groups (i.e., slopes) between mild pain and moderate-to-severe pain were also tested using a general linear model. * p<0.05, ** p<0.01, *** p<0.001
